# Electrospun Cellulose Acetate Nanofibers for Healthcare Products: Towards Sensing Pads for Endometriosis

**DOI:** 10.3390/polym18091036

**Published:** 2026-04-24

**Authors:** Theofilos Giannopoulos, Danai E. Prokopiou, Elias P. Koumoulos

**Affiliations:** 1 BioG3D, P.C., Agios Ioannis Rentis, 18233 Athens, Greece; 2 IRES—Innovation in Research & Engineering Solutions, Silversquare Europe, Square de Meeûs 35, 1000 Brussels, Belgium

**Keywords:** electrospinning, cellulose acetate, nanofibrous membrane, endometriosis, preventive medicine, toxicological safety

## Abstract

The need for reliable preventive medicine tools is growing, especially for diseases with long diagnostic delays, such as endometriosis, which can take several years to diagnose. In this context, cellulose acetate nanofibrous membranes were prepared via electrospinning, to create the absorbent core of a smart wearable in the form of a sanitary pad, intended to support electronic diagnostic devices. A multi-layered structure was opted for, with each layer acting in a specific way according to its position within the pad, regarding mainly absorbency and porosity. The membranes were ultralight and highly absorbent, with single membranes showing an absorbency of 20–70 times their initial weight, and multi-layered membranes 15–30 times. Morphological evaluation of the pad was used as the basis for the optimization of the fabrication parameters, while liquid absorption capacity confirmed the pad’s high absorbency. Additionally, chemical and toxicological assessments indicated in vitro biocompatibility of the pad. The potential of the electrospinning process in the fabrication of menstrual hygiene pads is shown by these results. Future studies should focus on the integration of smart devices within the pad, as well as their functionality and effectiveness.

## 1. Introduction

Smart wearables have been gaining popularity in recent years as a method of keeping track of a person’s health indicators, especially for cardiovascular health by providing metrics such as heart rate, oxygen levels and lastly an approximate estimation of blood pressure. Moreover, they are an important addition to preventive medicine, as they can indicate possible health issues before any physical symptoms occur [1]. However, while most current devices focus on cardiovascular and metabolic metrics, other areas, such as women’s health remain less covered. As a result, there is a growing need to focus on cases such as endometriosis. It is estimated that two-thirds of cases are diagnosed in women aged 20–35, with 10% affecting women under the age of 20. Endometriosis is a common condition, affecting 5–10% of women of reproductive age [2], with up to 50% of the diagnosed cases resulting in infertility. Endometriosis is a frequently painful condition in which endometrium-like tissue grows outside the uterus, with known hormonal, genetic and inflammatory differences [3]. Currently, laparoscopic surgery remains the gold standard for accurate diagnosis, highlighting the need for non-invasive diagnostic approaches. As a result, diagnosis is often delayed, potentially taking several years, with multiple doctor visits for a reliable result [4].

These challenges have led to the need to develop less invasive methods of diagnosis for such cases, with a promising approach being the fabrication of a smart sanitary pad via electrospinning, a versatile and simple technique that utilizes an electric field in order to stretch a polymer solution into ultrathin fibers, thus fabricating a non-woven, nanofibrous membrane [5]. It is a multifactorial process which relies on a multitude of parameters and material properties, such as solution viscosity, conductivity and surface tension, as well as process parameters such as applied voltage, working distance between the nozzle and collector and flow rate, while the temperature and relative humidity have also been reported to have an effect on the morphology and quality of the resulting fibers [6]. Of the solution parameters mentioned above, viscosity is one of the most critical parameters, as it affects both the solution’s capacity for electrospinning, as well as the morphology of the membranes [7].

Electrospinning has emerged as a fabrication technique for various applications, such as filtration [8,9,10], wound dressing [11,12,13], wound healing patches [14], drug delivery [15,16] and tissue engineering [17,18]. Its popularity has increased mainly in the biomedical and pharmaceutical sectors, for which a plethora of studies have been conducted [19]. The reason for this is its ability to create membranes with high surface area and porosity, as well as an indirectly tunable pore size [20]. Electrospinning can utilize a wide range of polymers as raw materials, including biologically sourced polymers, such as cellulose and its derivatives, chitin and chitosan, silk fibroin, collagen and gelatin [21,22,23,24]. Because of this, the increasing need for minimal environmental impact, especially regarding single-use products such as menstrual hygiene items, could also be addressed. Despite the progress, however, menstrual hygiene wearables have not received as much attention as other applications within the biomedical sector.

In recent years, the electrospinning of biopolymers has been widely reported on and recognized as a viable method of fabricating non-woven nanofibrous membranes for various applications. In the biomedical sector, the target has so far been applications such as tissue engineering, drug delivery, and wound dressings. In a study by Mousa et al. [25], the development of iron ions/nanoparticles within the cellulose acetate nanofibers was investigated for bone tissue engineering applications, while mats were evaluated for properties such as biocompatibility, biomineralization and structure. The study’s findings show that the formation of apatite-like structures can be facilitated by composite fibers of CA and iron acetate, improving biocompatibility. The electrospinning of biomaterials for wound healing patches that enable localized drug delivery has been thoroughly investigated. Huang et al. [26] successfully developed Janus nanofibers via parallel electrospinning of cellulose acetate and gelatin. As a result, both the biocompatibility and the mechanical stability of the resulting patch were improved. The nanofibers were then loaded with nanoparticles and nanomedicines, something that led to enhanced antimicrobial activity. Lastly, the incorporation of ZnO nanoparticles prolonged this effect.

Cellulose acetate (CA), a cellulose derivative that is produced by the acetylation of cellulose, has gained popularity for applications within the biomedical sector, due to properties such as biocompatibility and non-toxicity [27]. Moreover, it has been shown to be biodegradable [28,29], and while its degree of substitution plays an important role in its biodegradability, it is still considered a sustainable option. Lastly, its good absorbency when processed into porous fibrous structures [30] makes it a suitable candidate for applications where efficient moisture management is key, such as the absorbent core of a novel sanitary pad.

In the present study, the focus is on the development, optimization and characterization of the absorbent core of such a sanitary pad, through the electrospinning of cellulose acetate. The morphology, chemical composition, absorbency and in vitro safety of the produced membrane are evaluated in an effort to provide a comprehensive view of the process, while also ensuring the optimal properties of the final scaffold.

## 2. Materials and Methods

### 2.1. Materials

Cellulose acetate (CA) (molecular weight ~30kDa, 39.8% acetyl content, DS~2.5) was purchased from Sigma Aldrich, Taufkirchen, Germany. Dimethyl sulfoxide (≥99%) was purchased from Fisher Scientific, Loughborough, UK, and acetone (≥99.9%) was purchased from Honeywell, Charlotte, NC, USA.

### 2.2. Solution Preparation

The solvent system, DMSO/AC 25/75 *v*/*v*, based on the work reported by Neukirch et al. [31] was first prepared under mild stirring in room temperature and humidity (23–25 °C, 45–50%RH). The pre-weighed polymer powder was then gradually added into the solvent. An MN 226:90 × 115 mm weighing paper and a Kern KB240-3N scale (Kern, Grove City, OH, USA) (with a measuring accuracy of 0.001 g) were used for the powder measurement. The resulting mixture was initially stirred manually to ensure homogeneous distribution and subsequently stirred overnight at low RPM (170–180) at room temperature and humidity on a Jeio Tech Advanced Magnetic Stirrer with Hotplate (TS-18QG, Jeio Tech, Daejeon, Republic of Korea), until a transparent, homogeneous solution was obtained. Parafilm was used to cover the beaker and prevent solvent evaporation during stirring. The final solution was then loaded into a luer lock syringe for the electrospinning process. A 20 g stainless steel nozzle was used for the electrospinning process.

### 2.3. Electrospinning Process

The Spingenix SG10 electrospinning system (Spingenix, Palo Alto, CA, USA) was used. The electrospinning parameter window investigated in this study can be seen in Table 1. The nanofibrous membrane was deposited on a drum collector which was covered by aluminum foil and then collected at the end of each electrospinning session. The collector’s RPM was set at 400. After electrospinning, the membranes were allowed to dry overnight under a hood. The parameters were chosen based on the general trends provided by the literature.

The electrospinning trials that were chosen to move forward were designated as follows in Table 2.

These membranes served as the precursors to the fabrication of the multi-layered membranes that were the target of this work. These multi-layered membranes appear in Table 3.

### 2.4. Morphological Characterization

The morphological characterization of the produced membranes was examined using scanning electron microscopy (SEM) (Phenom ProX desktop, Thermo Fisher Scientific Inc., Waltham, MA, USA). The samples were sputter-coated with a conductive coating, using a SC7620 Mini Sputter Coater (Quorum technologies LTD, Lewes, UK). Sputter coating was performed for 90 s. ImageJ 1.54g was then used for the image analysis and fiber measurements, which was conducted on 60 fibers per sample using 20 from three distinct areas for each.

### 2.5. Chemical Characterization

Attenuated Total Reflectance Fourier-Transform Infrared Spectroscopy (ATR-FTIR) was performed on both the bulk material and the produced single-layered membranes (Table 1) to characterize the chemical structure and to detect any potential degradation following immersion of nanofibers in Dulbecco’s modified Eagle medium (DMEM). Spectra were acquired using an Agilent Cary ATR-FTIR 360 (Agilent Technologies, Santa Clara, CA, USA). The number of scans used for each sample was placed at 60.

### 2.6. Liquid Absorption Capacity

The liquid absorption capacity of membrane samples (5 cm × 5 cm) was evaluated by immersing the samples into the liquid medium (DI water), for 3 h and overnight based on the target application [32], following previously reported protocols [33]:

$$C\%=\frac{W_{1}-W_{0}}{W_{0}}100\%$$

$W_{1}$ ⟶ Weight after immersion.

$W_{0}$ ⟶ Weight before immersion.

C% ⟶ Absorption capacity as a percentage.

Before weighting, excess water was removed by allowing the samples to drain freely until dripping stopped.

### 2.7. Porosity

The apparent porosity of the membranes was determined using the dimensions and the initial weight of the samples employed in the absorption capacity tests. The porosity was calculated using the following equation, as reported previously [34]:

$$P\%=\left(1-\frac{\mathrm{Apparent}\;\mathrm{density}}{\mathrm{Bulk}\;\mathrm{density}}\right)100\%$$

where the apparent density is calculated by the following:

$$d=\frac{\mathrm{Weight}\;\mathrm{before}\;\mathrm{immersion}}{\mathrm{Length}\times \mathrm{WIdth}\times \mathrm{Thickness}}$$

The bulk density value was obtained from the material characteristics given by the supplier [35].

### 2.8. Thickness Measurements

The thickness of the electrospun membranes was measured at three different points, using a micrometer with a digital display (Yato YT-72305, Yato, Baibu, China—0–25 mm).

### 2.9. Wettability Assessment

The wettability of the samples was assessed by the drop method with deionized water (DI) water as the liquid medium, using the Ossila Contact Angle goniometer and its associated analysis software (Ossila, Sheffield, UK). A Hamilton microsyringe was used for the protocol and three droplets for each specimen were tested.

### 2.10. Toxicological Testing Protocols for Safety Assessment of Electrospun Membranes

#### 2.10.1. Preparation of Electrospun Membranes Before In Vitro Analysis

Electrospun membranes were cut into circular disks (0.5 cm diameter), and each membrane was sterilized under UV light for 30 min. The sterilized discs were then placed into a 96-well cell culture plate for, following the method described by Basak et al. [36]. Cells were incubated with the test materials for 24 h. After exposure, the cells were processed according to the preferred protocols.

#### 2.10.2. Cell Culture and Cytotoxicity Study of Electrospun Membranes

To assess the effects of the electrospun membranes on the skin, human dermal fibroblasts Hs27 (CRL-1634, ATCC) were cultured in direct contact with membranes. These cells were obtained from the American Type Culture Collection (ATCC, Manassas, VA, USA) and handled in accordance with the manufacturer’s instructions. These types of cells were cultured in Dulbecco’s modified Eagle medium (DMEM) supplemented with 10% (*v*/*v*) fetal bovine serum (FBS) and 1% penicillin–streptomycin antibiotics to prevent bacterial growth. Cells were maintained at 37 °C in a humidified atmosphere containing 5% CO_2_ (Thermo Scientific Forma Steri-Cycle i160 CO_2_ incubator, 37 °C, 5% CO_2_).

The MTT assay (3-(4,5-dimethylthiazol-2-yl)-2,5-diphenyltetrazolium bromide, Sigma-Aldrich, St. Louis, MO, USA) is used to measure the cellular metabolic activity when the cells come into direct contact with nanofibrous membrane. Briefly, 5 × 10^3^ cells were seeded in a 96-well plate and incubated at 37 °C to grow overnight. After 24 h, the sterilized nanofibrous membranes were incubated with the cells for an additional 24 h. Subsequently, the culture medium and nanofibrous were removed and 100 μL of MTT solution (5 mg/mL in PBS) was added to each well. The plate was then incubated at 37 °C for 2–3 h, where dark purple formazan crystals precipitated as evidence of live cell presence. The plate was incubated in the dark on an orbital shaker for 15 min. The absorbance was measured at OD = 590 nm using a plate reader (FLUOstar^®^ Omega plate reader, BMG Labtech, Ortenberg, Germany), within 1 h.

#### 2.10.3. Assessment of Intracellular Reactive Oxygen Species Production

To assess intracellular production of reactive oxygen species (ROS), Hs27 cells were seeded at a density of 5 × 10^3^ in a 96-well plate and allowed to attach overnight at 37 °C in a humidified 5% CO_2_, aiming for approximately 70% confluency. After 24 h, the cells were exposed to the sterilized nanofibers for an additional 24 h. Each condition was tested in triplicate. In addition to untreated cells as control cells, a positive control was included in parallel to validate the assay. Pyocyanin, a cell-permeable compound capable of redox cycling, is included as a positive control for ROS generation.

After the exposure period, ROS levels were measured using a commercial detection kit (DCFDA, Item No. 601520, Cayman Chemical, Ann Arbor, MI, USA), following the manufacturer’s protocol. The cell-permeable probe is deacetylated by intracellular esterases to H2DCF, which is subsequently oxidized by ROS to the highly fluorescent compound DCF. Fluorescence intensity, which correlates with intracellular ROS level, was measured using a plate reader (FLUOstar^®^ Omega plate reader, BMG Labtech, Ortenberg, Germany) at an excitation/emission (Ex/Em) wavelength of 495/529 nm. ROS levels were expressed as a percentage relative to untreated control cells, which were set at 100%.

#### 2.10.4. Inflammatory Response Assessment

To assess the potential inflammatory response induced by electrospun membranes, Hs27 cells were seeded in 12-well plates and allowed to adhere overnight. After 24 h, the cells were exposed to the nanofibers for 24 h, under the standard culture conditions (at 37 °C in a humidified atmosphere with 5% CO_2_). After the exposure period, the release of the proinflammatory cytokines interleukin 18 (IL-18) (OriGene Human IL-18 ELISA Kit, OriGene, Rockville, MD, USA) and tumor necrosis factor-alpha (TNF-a) (Abnova TNF-α (human) ELISA Kit, Abnova, Taipei City, Taiwan) into the cell culture supernatant was quantified using an enzyme-linked immunosorbent assay (ELISA), according to the manufacturer’s protocol.

Calibration curves for IL-18 and TNF-α were established using the standard concentrations included in each ELISA kit. The levels of cytokines in the collected samples were then determined by applying the corresponding linear equation from each curve, and the results were expressed in pg/mL. For each experimental group, mean values were calculated and compared with those of the untreated control to assess the inflammatory response.

### 2.11. In Vitro Degradation Study

Electrospun membranes were cut into circular specimens (15 × 15 mm) for in vitro degradation experiments. The in vitro degradation behavior was assessed in DMEM. Each sample was placed in a 24 well plate containing 3 mL of DMEM and incubated at 37 °C and under 5% CO_2_ for 48 h [37]. After 48 h, the samples were collected and rinsed thoroughly with distilled water and dried for subsequent characterization (FT-IR and SEM).

### 2.12. Statistical Analysis

The results are expressed as the mean ± standard deviation (SD). Statistical comparisons were conducted using single-factor (one-way) ANOVA. Statistical significance was defined at three levels: *p* < 0.05 (*), *p* < 0.01 (**), and *p* < 0.001 (***). Each experimental condition was tested in at least duplicate (n = 2) or triplicate (n = 3).

## 3. Results

### 3.1. Chemical and Morphological Characterization

#### 3.1.1. Chemical Characterization—FTIR

**Figure 1:**
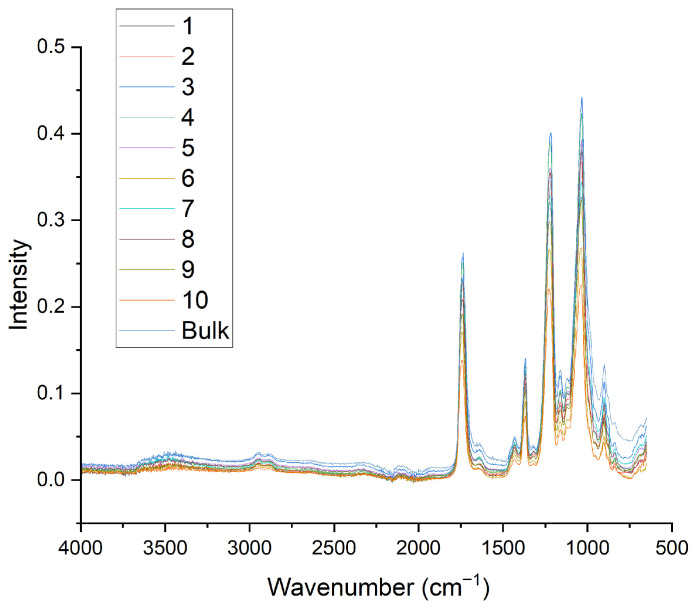
FTIR comparison between the fabricated CA membranes and bulk CA in powder form. The numbering of the samples is according to Table 2.

FTIR was used as a first step in the evaluation of the membranes, in order to assess the chemical structure of the membranes and if there were any changes after they were dried. Figure 1 shows the spectra of 10 different electrospun membranes, from Table 1 (referring to single layers), which describes the parameters used for the single layers, juxtaposed with the spectrum of bulk CA.

Both the bulk CA and the electrospun membranes show four prominent peaks, which are characteristic of cellulose acetate. At 1738 cm^−1^, the stretching vibrations of C=O bonds are observed. The next peak, at 1366 cm^−1^, shows C-CH_3_, while the peak at 1216 cm^−1^ is indicative of C-O-C bond stretching. Lastly, the peak at 1032 cm^−1^ shows the stretching vibrations of C-O bonds [38]. Interestingly, the membranes show peaks with lower intensity compared to those of the bulk material. This may be a result of the porous structure of the membranes, which can reduce effective contact with the ATR-FTIR crystal. The membranes that were characterized using FTIR are detailed in Table 3.

#### 3.1.2. Morphological Characterization

**Figure 2:**
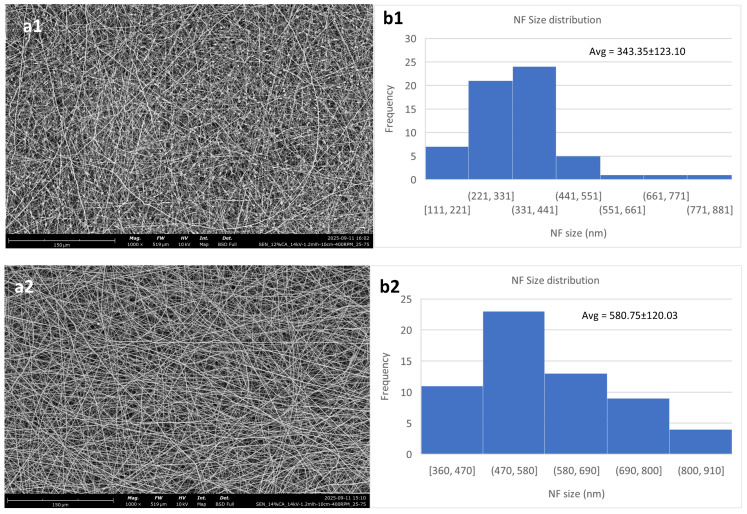

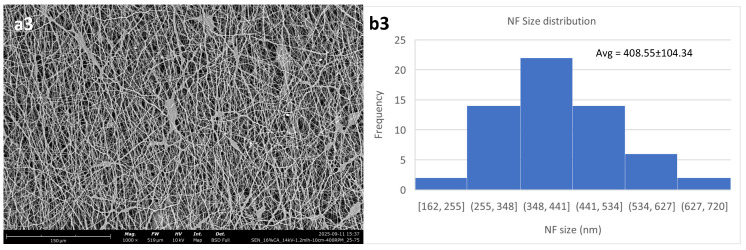
Electrospun cellulose acetate nanofibrous meshes. The constant parameters for all are DMSO/acetone 25/75; high voltage: 14 kV; flow rate: 1.2 mL/h; N-C distance: 10 cm; and collector RPM set at 400. (**a1**,**b1**) 12% *w*/*v*; (**a2**,**b2**) 14% *w*/*v*; (**a3**,**b3**) 16% *w*/*v*. (**a**) shows the fibers, while (**b**) shows the NF size distribution and average.

Figure 2 shows the effect of polymer concentration on the nanofibrous meshes. Evidently, the 12% *w*/*v* consistently produced membranes of lower quality, with a higher content in defects than the other two. This is in agreement with Kramar et al. [39] who showed that the optimal polymer concentration regarding CA for electrospinning is between 14 and 17% *w*/*v*.

The overall optimal result for these parameters is taken from the 14% *w*/*v* solution, even though it also has the largest fibers. It exhibits the lowest standard deviation, while also showing minimal content in defects.

**Figure 3:**
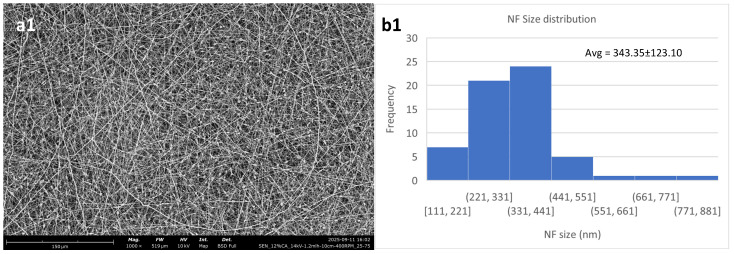

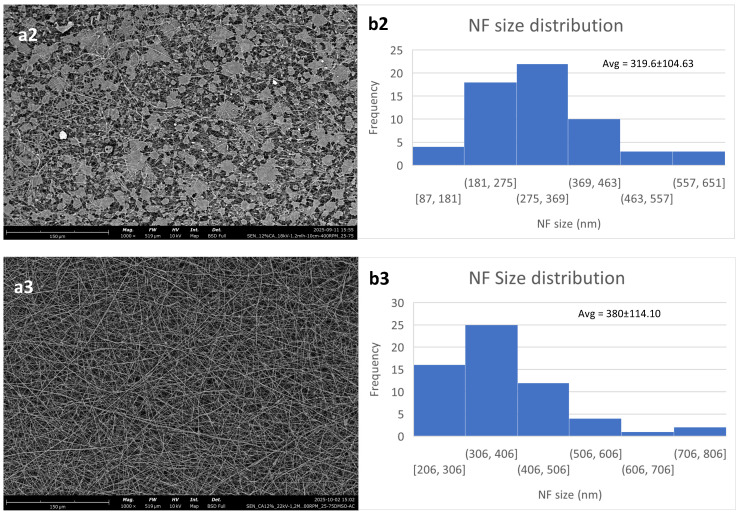
Electrospun nanofibers of a 12% *w*/*v* cellulose acetate solution in DMSO/acetone 25/75 *v*/*v*. (**a1**,**b1**) 14 kV, 1.2 mL/h, 10 cm, 400 RPM. (**a2**,**b2**) 18 kV, 1.2 mL/h, 10 cm, 400 RPM. (**a3**,**b3**) 22 kV, 1.2 mL/h, 10 cm, 400 RPM.

The 12% *w*/*v* solution shows a high content of beads on its fibers, while also having the least size homogeneity. This is due to the concentration being lower than optimal, which has been reported to lead to beaded fibers [40]. It should be noted that electrospinning of this solution resulted in non-uniform fiber deposition. The 12% *w*/*v* solution also showed a high concentration of beads when electrospun at 22 kV, while an effort of electrospinning at 18 kV led to the formation of a porous film-like structure instead of a nanofibrous membrane. Figure 3 shows that there is minimal difference between 14 kV and 22 kV for the 12% *w*/*v* solution, except for a slight increase in the mean diameter and a decrease in the standard deviation, while for the 18 kV voltage setting, the membrane was morphologically suboptimal.

**Figure 4:**
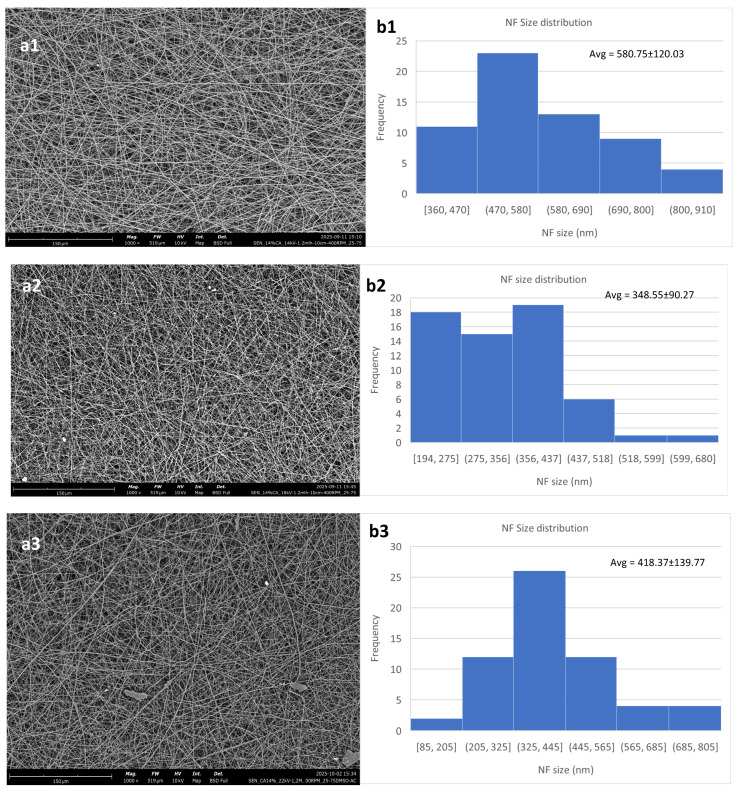
Cellulose acetate nanofibrous membranes created with a polymer concentration of 14% *w*/*v*. (**a1**,**b1**) at 14 kV, (**a2**,**b2**) at 18 kV and (**a3**,**b3**) at 22 kV. The rest of the parameters were constant, flow rate at 1.2 mL/h, N-D distance at 10 cm and collector RPM at 400.

For the 14% solution, the results are in general more consistent. In all three voltages used, the nanofibrous membrane has a low content of defects. The fiber size shows an initial decrease with the increase in the voltage from 14 to 18 kV and then starts increasing again. This behavior has been reported by Jalal et al. [41], who show that above the optimal voltage range, which for this composition is shown to be between 14 and 18 kV, the fiber sizes start increasing again. This may be attributed to the increased rate of solvent evaporation or the increased amount of polymer that is extruded as the voltage surpasses a critical value. Figure 4 shows this behavior, as well as SEM micrographs of the three different membranes created.

**Figure 5:**
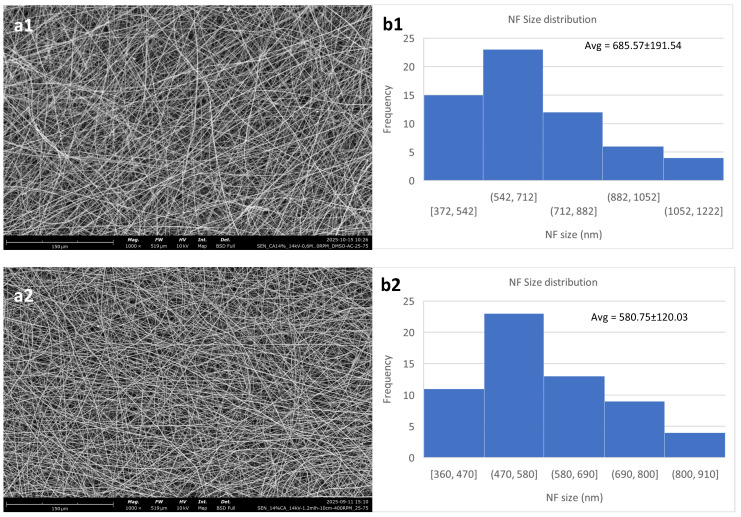

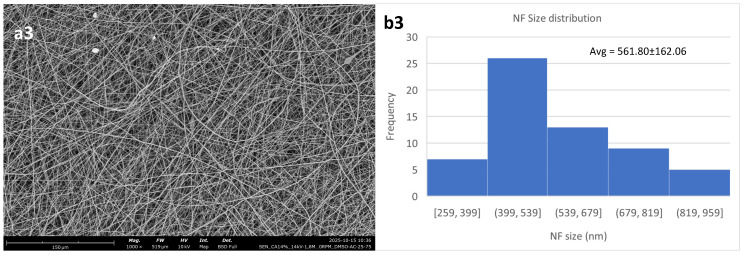
Membrane comparison based on flow rate, with high voltage, working distance and collector RPM kept constant at 14 kV, 10 cm and 400 RPM, respectively. (**a1**,**b1**) 0.6 mL/h, (**a2**,**b2**) 1.2 mL/h and (**a3**,**b3**) 1.8 mL/h.

The 14% solution was delved deeper into, given that it showed the most promising results in terms of reliable fabrication at lower voltages. A comparison between the results with different flow rates was carried out. The morphology of these trials can be seen in Figure 5. As can be seen, lower flow rates can lead to a higher fiber size.

At the same time, the size becomes less homogeneous than the one obtained at 1.2 mL/h. However, the morphology of the membrane created the low flow rate setting appears to be pristine, with no content in defects, similar to the one obtained at 1.2 mL/h.

On the other hand, when the flow rate increases, the fiber size is reduced. While the standard deviation is similar to that of the membrane created at 0.6 mL/h, and higher than the one created at 1.2 mL/h, this membrane also showed the presence of beads and polymer aggregations—something that could be explained by the fact that more polymer was extruded within the same time interval as the previous two and at the same time there was less time available to allow for optimal solvent evaporation [42]. While the general trend shows a decrease in fiber size while the flow rate increases, the difference between 1.2 mL/h and 1.8 mL/h is not significant. At the same time, the standard deviation of the low and high flow rate settings is significantly higher than that of the middle setting, along with the higher presence of defects in the membrane created at the higher flow rate, led to the conclusion that the middle flow rate setting would be optimal within the scope of this work.

**Figure 6:**
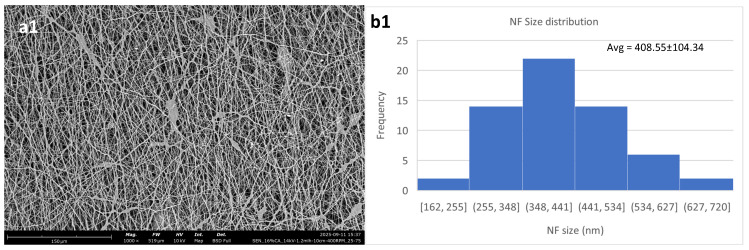

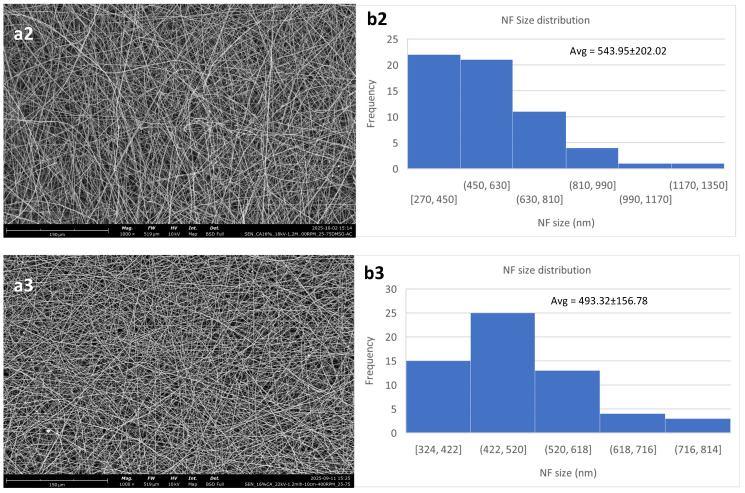
Electrospun membranes of 16% *w*/*v* cellulose acetate in DMSO/acetone 25/75. (**a1**,**b1**) 14 kV, 1.2 mL/h, 10 cm, 400 RPM. (**a2**,**b2**) 18 kV, 1.2 mL/h, 10 cm, 400 RPM and (**a3**,**b3**) 22 kV, 1.2 mL/h, 10 cm and 400 RPM.

While the 16% solution also shows a beaded structure for the lower-voltage setting, this is remedied by the increase in the high voltage, as reaching 22 kV appears to remove these defects from the surface. This can be explained by the increased polymer concentration, which can lower solution conductivity, as shown by the work of Majumder et al. [43], leading to the need for a higher applied voltage in order to create a membrane of optimal quality, which can be seen in Figure 6, where the membrane obtained by 14 kV is compared to the one obtained by 22 kV. At the same time, the 16% solution gives a membrane with higher homogeneity than that of the 12% solution, which is indicative of the effect of the polymer concentration on the process.

In the 16% solution, it is apparent that a high content in defects can also lead to a lower average fiber diameter.

Figure 6(a3) shows the membrane obtained from a 16% *w*/*v* solution with a high voltage of 22 kV. Similarly to the 14% of the previous section, this one also has minimal defects; however, its standard deviation has increased compared to the one obtained at 14 kV. This could mean that the higher the polymer concentration, the higher the critical voltage range, in regard to the morphology of cellulose acetate membranes.

From the SEM images shown above, it is apparent that the voltage and the polymer concentration play an important role in the homogeneity of fiber sizes, as the three cases show differing behaviors with the increase in voltage.

**Figure 7:**
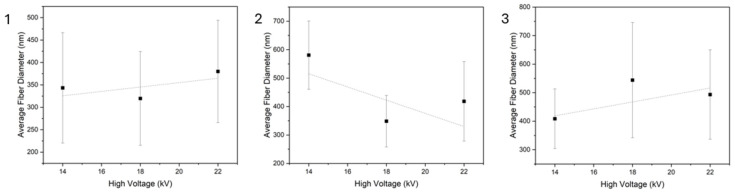
Average fiber diameters for the three different compositions in relation to the high-voltage setting. (**1**) 12% *w*/*v*; (**2**) 14% *w*/*v*; (**3**) 16% *w*/*v*.

Figure 7 shows the average fiber diameters along with their standard deviations taken in relation to the polymer concentration used and the applied voltage. The 12% and 14% solutions follow opposing trends. This behavior may be attributed to the 12% *w*/*v* being lower than the optimal concentration for cellulose acetate, leading to insufficient chain entanglement, something that can lead to unstable electrospinning at lower voltages [44], while raising the voltage led to the mitigation of this effect.

As previously mentioned, the 14% concentration was shown to be the optimal concentration within the scope of this work, and this is also depicted in the overall low standard deviations that it shows for 14 and 18 kV. At 22 kV, the applied voltage reaches a level that is too high for this composition, leading to the process becoming unstable due to the higher electrical field that is applied [45].

Lastly, the 16% initially shows an increase in standard deviation and then it decreases again, as the applied voltage increases, while showing the lowest standard deviation at 14 kV as well, similar to the 14% solution. In relation to the morphology that these exhibit, this could be explained by the voltage of 18 kV being enough to form beadless fibers, while at the same time being at the low threshold of the optimal high-voltage window, meaning it could cause jet instability—something that would lead to a higher standard deviation.

Overall, the 12% and 16% *w*/*v* solutions show an upward trend for their fiber diameters in relation to applied voltage, while the 14% shows a downward trend.

Table 4 shows the average fiber diameter and standard deviation values for each case.

These membranes were the precursors for the later multi-layered structures. Because of the overall better results (morphology and size homogeneity) that the 14% *w*/*v* solution gave at lower voltages, greater emphasis was placed on it.

### 3.2. Liquid Absorption Capacity and Porosity

**Figure 8:**
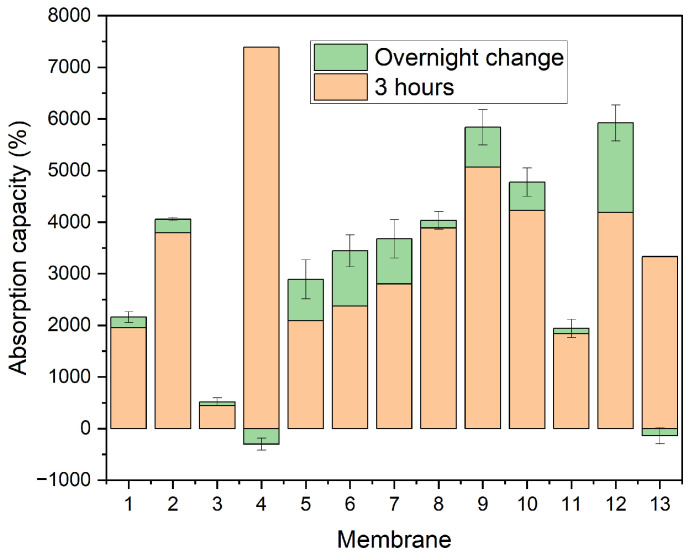
Liquid absorption capacity for the cellulose acetate nanofibrous membranes.

Figure 8 shows the absorption capacity of several trials. It shows that the absorption capacity is mostly at 10 to 40 times the dry weight of the samples, while some of them exceed that.

From Figure 8, it is evident that most of the created membranes exhibit good absorbency, starting at 20 times their initial weight and reaching up to 70. The only exception to this is Membrane No. 3, which can be explained by its comparatively lower porosity, which is presented next. Moreover, the membrane that was created using this set of parameters, instead of a soft membrane, gave a tougher, film-like consistency.

Membrane No. 4 appears to lose a small percentage of DI water during the overnight timepoint; however, it still exhibited the highest absorbency of the electrospun membranes that were tested. The above electrospun membranes are detailed in Table 5.

Table 6 shows the absorbency results and their respective standard deviations in percentage form. While there are instances where the SD is high, reaching up to 21.5%, this appears to be prevalent in the membranes that have been previously shown to be of suboptimal quality.

Each of the samples showed a high absorption already at 3 h, and built up from that to various degrees, during overnight immersion.

**Figure 9:**
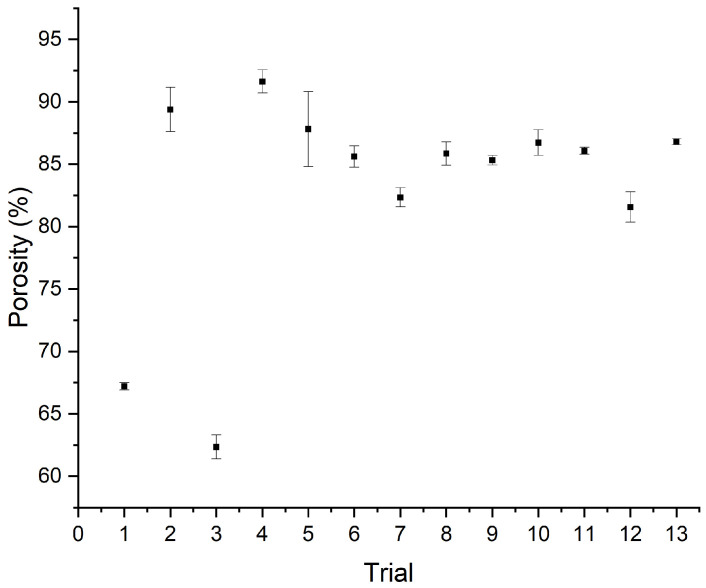
Porosity measurements for the cellulose acetate nanofibrous membranes.

Based on the dimension measurements conducted during these tests, the calculation of the porosity of the membranes was also possible. Figure 9 shows that the porosity was mostly at 80–90%, which is within the normal range reported, with 90% being at the higher limit [46,47]. For the apparent porosity estimation, the measurement of the thickness of each membrane was necessary. The values for both thickness and apparent porosity appear in Table 6.

The porosity for Membranes No. 1 and 3 is evidently lower than that of the rest of the samples, and outside the general porosity range that has been reported for CA membranes electrospun under similar conditions, which can also help to explain their comparatively lower absorption capacity.

The rest of the membranes show porosities mainly between 82% and 88%, which is in line with the reported values for cellulose acetate membranes [48], although they have been shown to reach up to 95% in extreme cases.

**Figure 10:**
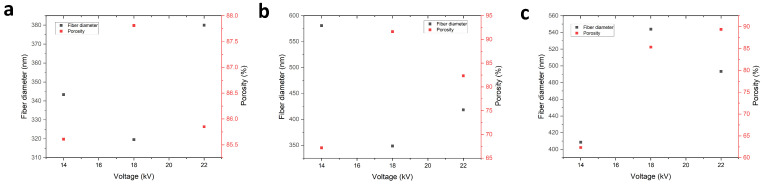
Porosity as related to fiber diameter for all three polymer concentrations. (**a**) 12% *w*/*v*; (**b**) 14% *w*/*v*; (**c**) 16% *w*/*v*.

It has been generally reported that the fiber size has a significant impact on the porosity and pore size of the membranes [49]. The fiber diameter of the samples appears to have a negative impact on the overall porosity of the membranes within the scope of this work. Figure 10 showcases this for the three different consistencies, in the three different voltage settings used, where finer diameters generally lead to higher porosities. This could explain the highly different porosity of the first sample, as it has the largest average fiber size when compared to the other two cases for the 14% consistency when the voltage changes.

#### Multi-Layered Membranes

**Figure 11:**
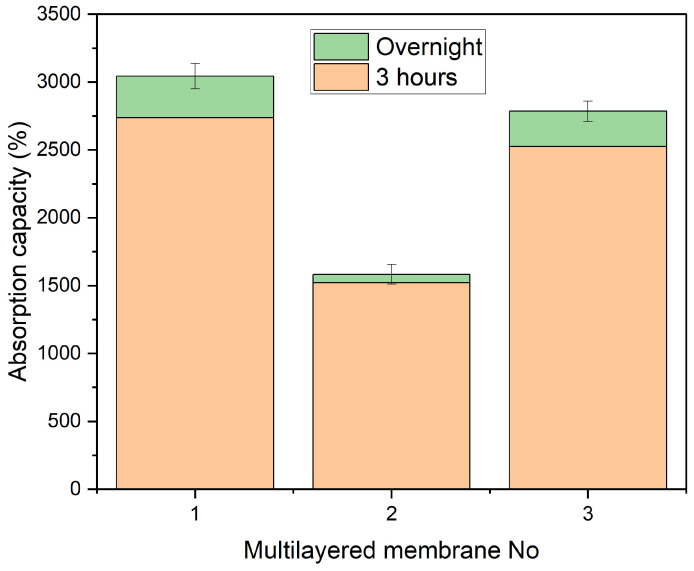
Multi-layered membranes absorption capacity. The first and third samples exhibit a higher overall capacity, while the second sample absorbed the DI water faster than the other two.

From Figure 11 it is apparent that the multi-layered samples, even though they are comprised of the individual layers that are shown in this work, have a reduced absorption capacity. A likely answer as to why this is happening could be that the layers that are added onto the earlier ones close previously open pores.

**Figure 12:**
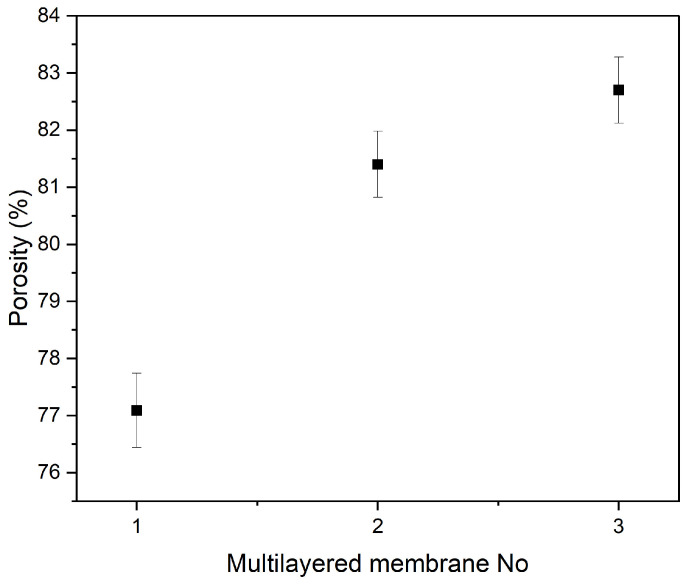
Porosity measurements of the multi-layered samples. The third layer has the highest mean porosity and the first the lowest.

It has also been shown that the porosity of the final membrane depends on the time that the process is running. Namely, the longer the process runs, the lower the porosity [46]. However, the multi-layered samples also show more consistent results. Figure 12 shows the porosity measurements for the three multi-layered samples. Three measurements were taken for each membrane, based on the measurements conducted on the samples that were immersed.

Similarly to the single electrospun membranes, prior to the estimation of the apparent porosity, the thickness was measured. These values appear in Table 7 and Table 8.

The difference between the three samples can be explained by the thickness they exhibit. It is important to note that Membrane No. 3 consists of three layers, while Membranes No. 1 and 2 consist of four.

Given the above results, it is evident that a fourth layer would be obsolete within the scope of this work, as it does not significantly increase absorbency. A three-layered membrane was therefore decided, as it could save both material and time without compromising the functionality of the structure.

### 3.3. Wettability Assessment

The multi-layered membranes show better wettability overall, while the single layers themselves show a more hydrophobic behavior. This could be attributed to an initial Cassie–Baxter like behavior that the membranes could exhibit due to air entrapment that prevents the invasion of the liquid within the electrospun membrane [50].

**Figure 13:**
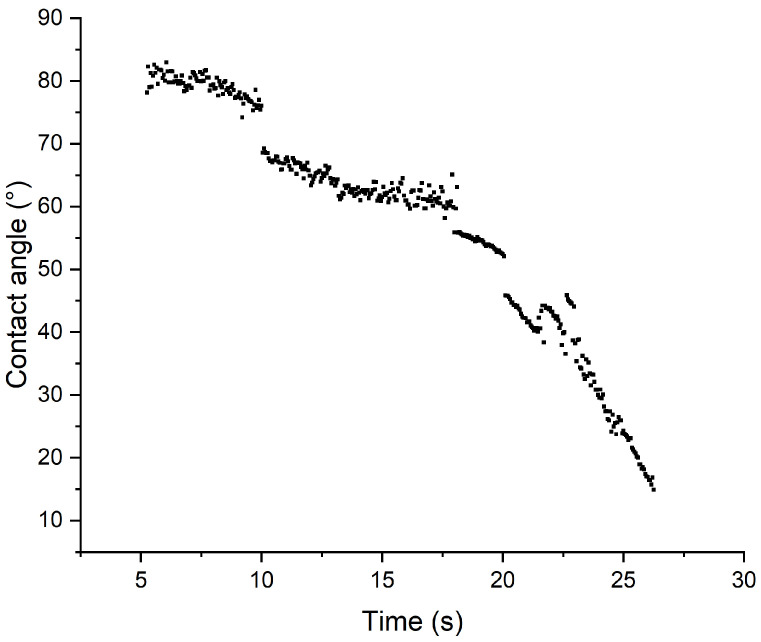
Droplet absorption rate for ML2.

**Figure 14:**
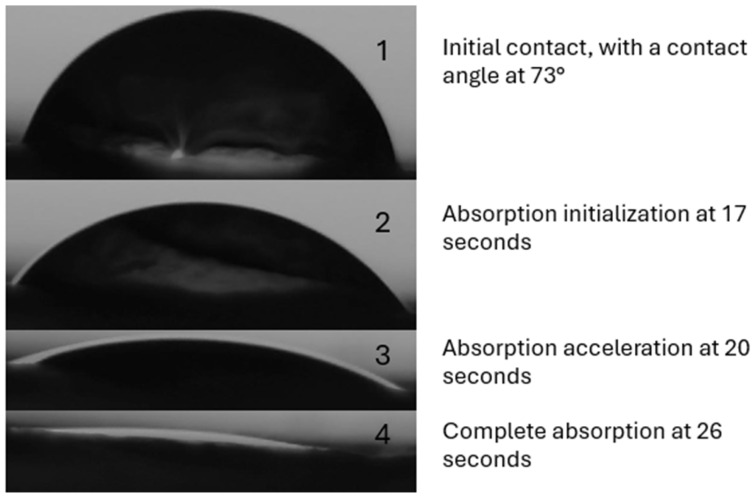
Depicted absorption of DI water on ML2.

As previously stated, ML2 showed the best wettability of the three, showing a complete absorption of a droplet within 30 s, as shown in Figure 13, while Figure 14 shows the pictures taken by the analysis software at each step.

**Figure 15:**
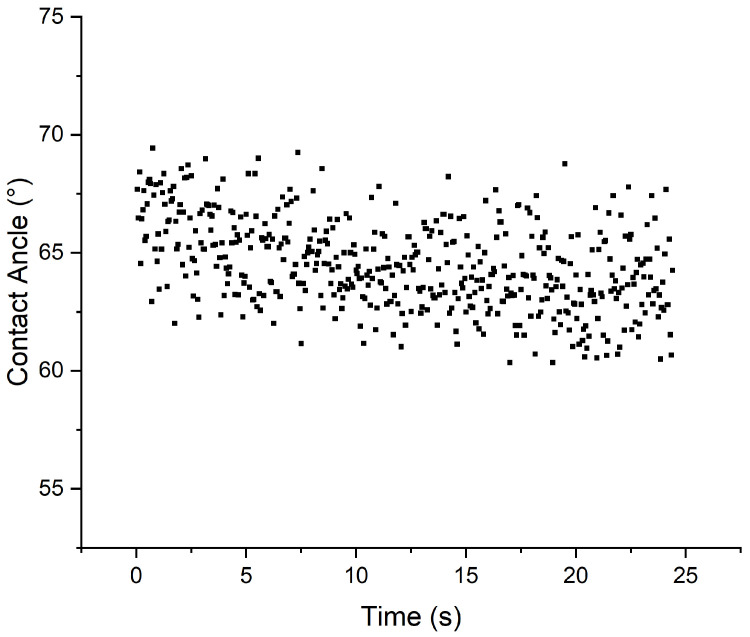
Contact angle behavior for the ML3 sample.

In contrast, for ML1 and ML3, while hydrophilicity was observed, the absorption rate was much slower. Figure 15 shows the behavior of ML3 within the same timeframe. The contact angle of the droplet is stable at about 65°. The same behavior can be observed by the ML1 sample.

Table 9 shows the initial contact angles for the three multi-layered samples, taken as an average of three droplets.

### 3.4. Toxicological Assessment

After optimizing the electrospinning parameters, we selected the final electrospun membranes for biological evaluation. These included multi-layered membranes (ML-1, ML-2, and ML-3) and membranes prepared from a DMSO/AC (25:75) solvent system containing 14% and 16% polymer (Nos. 1 and 2 according to Table 2), which exhibited consistent fiber formation, as well as a 12% (No. 5 according to Table 2) polymer membrane that displayed the least well-defined morphology. The selected membranes were used to investigate whether the structural differences influence cellular response and to determine their overall biocompatibility profile.

#### 3.4.1. Cytotoxicity and Reactive Oxygen Species Production Induced by Electrospun Membranes

**Figure 16:**
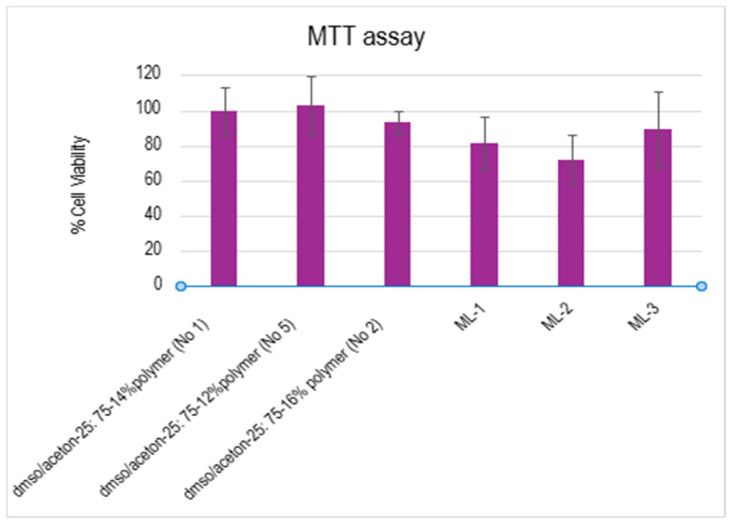
Cytotoxicity of the electrospun membranes against Hs27 cell lines using MTT assay for 24 h incubation. Data are presented as the mean ± SD (n = 3). Statistical analysis performed using one-way ANOVA (single factor). No significant differences were observed between groups.

Τhe cytotoxicity of the tested electrospun membranes were evaluated using MTT assay. All experiments were performed in triplicate (n = 3), and the results are presented as the mean ± standard deviation (SD). As we can observe (Figure 16), all tested membranes exhibited cell viability above 70%. These results indicate that the tested membranes did not induce significant cytotoxic effects and can be considered biocompatible under the examined conditions [51]. This reduced viability values in multi-layers are more likely related to structural parameters rather than material-related toxicity.

**Figure 17:**
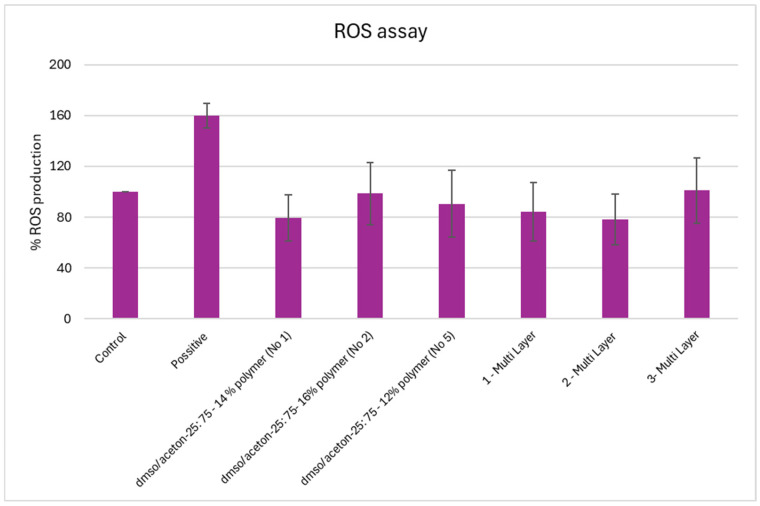
ROS production (%) in Hs27 cells treated with nanofibers. Data represent the mean ± SD (n = 3). Statistical analysis was performed using one-way ANOVA (single factor). No significant differences were observed between groups.

The production of intracellular reactive oxygen species in cells was evaluated with a commercial fluorometric assay in the Hs27 fibroblast cell line, after 24 h membrane exposure. All measurements were performed in triplicate (n = 3), and data are expressed as the mean ± SD. The positive control caused a clear increase in ROS, while untreated cells (control) showed baseline levels. ROS production remained at baseline levels across all tested membranes and did not differ significantly from the untreated control (*p* > 0.05) (Figure 17). Although the differences were not statistically significant, the slightly lower mean values observed in some groups could be related to the hydrophilic character of cellulose acetate, which favors a stable cell–material interaction, or to the nanofibrous architecture acting as a mild physical interface between the cells and the surrounding environment.

The fact that ROS levels did not increase could be related to the intrinsic biocompatibility of cellulose acetate. Previous studies have reported that CA nanofibers support cell growth without causing oxidative stress, likely because of their stable chemical structure and hydrophilic surface, which provide a gentle environment for cells [52,53]. In the context of menstrual hygiene pads, where materials must remain in contact with sensitive tissues for extended periods, the absence of ROS is one factor that supports the safety and biocompatibility of the electrospun membrane structures.

#### 3.4.2. Cytokine Release in Hs27 Cells Following Contact with Electrospun Membranes

To examine whether the electrospun membranes could induce any skin-related sensitivity upon direct contact, we assessed the release of the proinflammatory cytokines IL-18 and TNF-a in the cell culture media following 24 h exposure of the Hs27 cell line to the electrospun membranes. Cytokine measurements were performed in duplicate (n = 2), and the results are presented as the mean ± SD. Il-18 is commonly associated with early skin irritation responses, as it is released by keratinocytes and other skin-related cells upon inflammatory activation [54]. For this reason, it is often used as an indicator of subtle irritation effects. In parallel, TNF-α was selected as a broader marker of inflammatory activity. This cytokine is mainly produced by activated monocytes and macrophages, although several other cell types may also contribute under inflammatory conditions. Altered TNF-α signaling has been linked to a wide range of inflammatory disorders, highlighting its relevance in assessing the biocompatibility of materials intended for skin contact [55].

**Figure 18:**
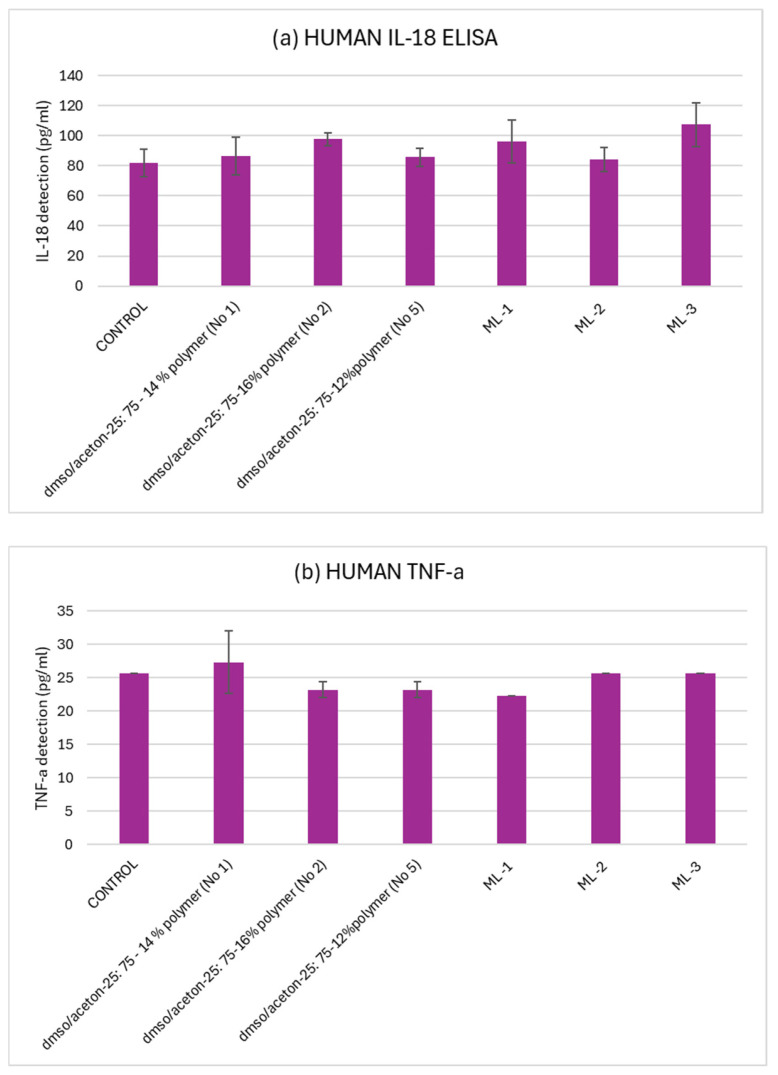
IL-18 (**a**) and TNF-a (**b**) levels after direct contact with different types of electrospun membranes (n = 2 per group). No significant differences were detected through one-way ANOVA (*p* > 0.05), indicating the absence of an early inflammatory response.

As shown in Figure 18, the detected IL-18 and TNF-a concentrations in the electrospun membranes-treated samples both remained close to the untreated control. Statistical analysis using one-way ANOVA confirmed that no significant differences were observed between the treated types of electrospun membranes (*p* > 0.05). These findings support that the materials did not trigger early inflammatory or skin-sensitivity responses under the tested conditions.

### 3.5. Morphological and Structural Changes Observed During In Vitro Degradation

**Figure 19:**
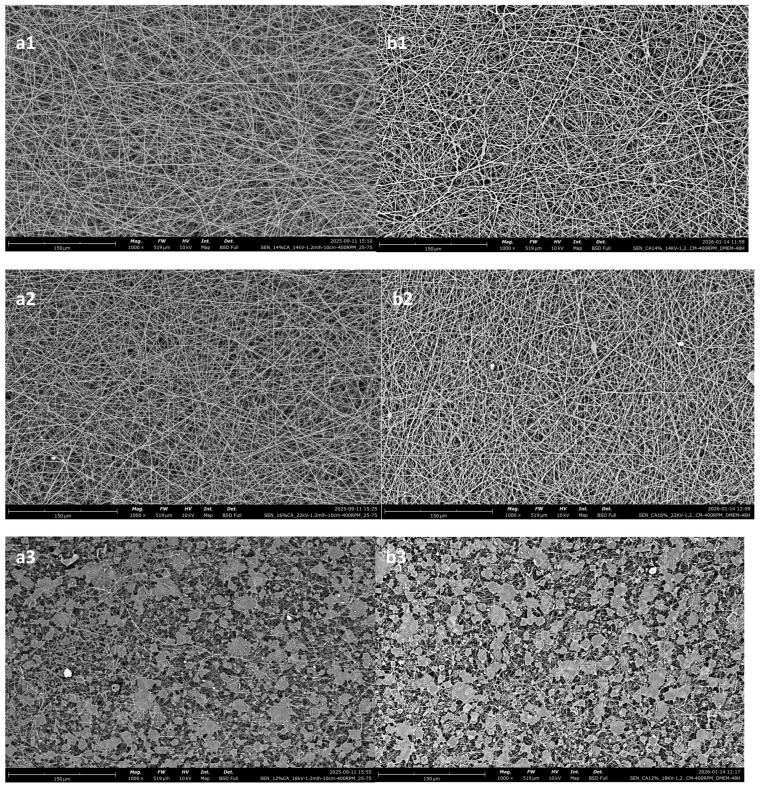
Single-layered electrospun membranes (1, 2 and 5) before (**a1**–**a3**) and after (**b1**–**b3**) incubation in DMEM. Where (**a1**,**b1**) 14% *w*/*v*, (**a2**,**b2**) 16% *w*/*v* and (**a3**,**b3**) 12% *w*/*v*.

To check whether the electrospun membranes changed during the in vitro degradation period, the degradation process was studied before and after 48 h immersion in DMEM and a morphological study of the nanofibers was carried out by scanning electron microscopy (SEM). The SEM images did not show any obvious changes in the morphology of nanofibers; there were no cracks. While the single-layered membranes exhibited no swelling, the multi-layered ones did (Figure 19).

**Figure 20:**
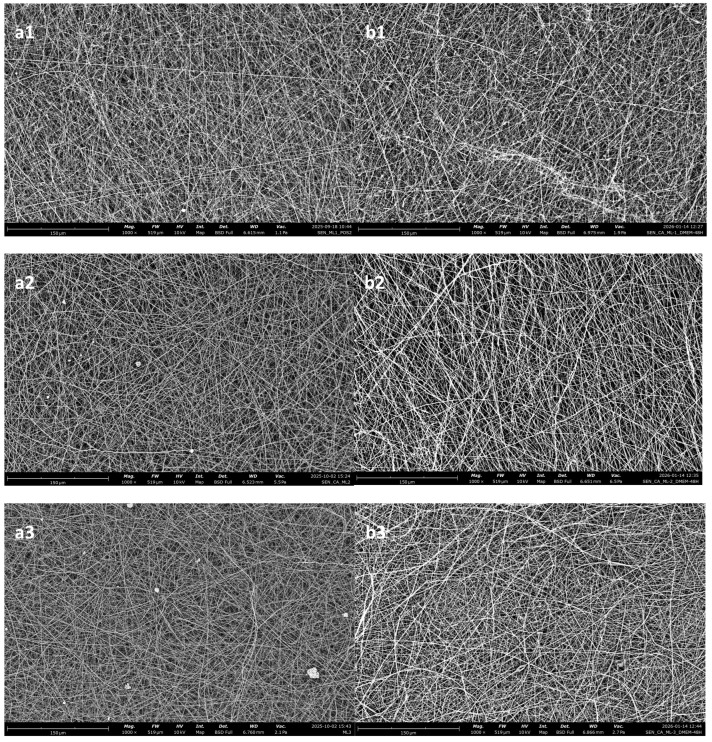
Multi-layered electrospun membranes before (**a1**–**a3**) and after (**b1**–**b3**) incubation in DMEM. Where (**a1**,**b1**) Multilayered membrane 1, (**a2**,**b2**) Multilayered membrane 2 and (**a3**,**b3**) Multilayered membrane 3.

Figure 20 shows the state of the multi-layered membranes before and after incubation in DMEM.

For both the single-layered electrospun membranes and for the multi-layered ones, there appears to be no significant visual degradation.

**Figure 21:**
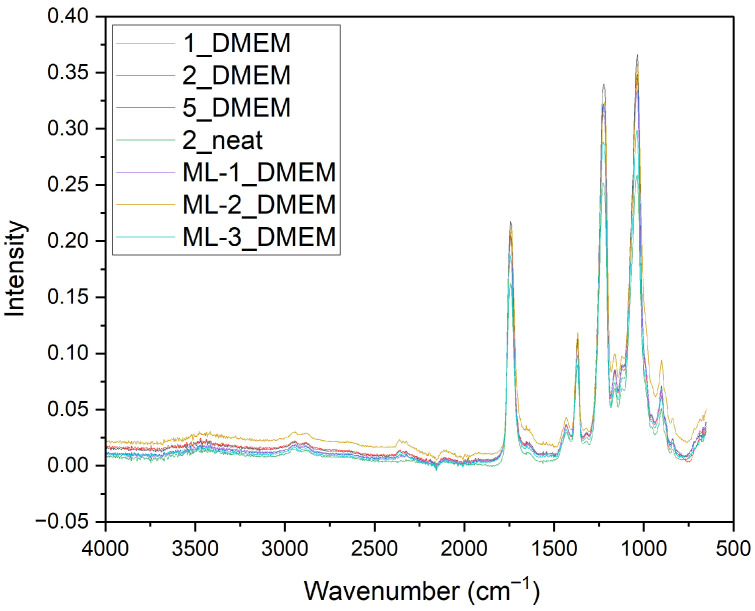
FTIR comparison between a neat electrospun membrane (Membrane No. 2) and the membranes after incubation in DMEM.

The FTIR spectra (Figure 21) also matched well with the initial material. This showcases the chemical stability of cellulose acetate electrospun membranes, as no apparent chemical structural degradation occurs after the incubation.

## 4. Discussion

In this study, the fabrication of non-woven nanofibrous membranes via the electrospinning of cellulose acetate was investigated, with the aim of creating an absorbent core for smart sanitary pads. The membranes’ high liquid absorption capacity, a critical property for the intended application, was demonstrated during this study, but also their non-toxicity and biocompatibility, properties that are desired in the broader spectrum of the biomedical field.

The three different voltage settings used for the three different polymer concentrations were utilized to create single-layered electrospun membranes, showing different behaviors for each regarding the results that the trials yielded when taking into account the observed morphology. While the optimal voltage setting for the 14% *w*/*v* solution is observed to be at 14–18 kV, this is not the case for the 16% *w*/*v* solution, which is shown to require a higher voltage in order to produce membranes of optimal quality. This may be attributed to the increased polymer concentration, which in turn decreases the conductivity of the solution, thus requiring a higher voltage to achieve the fabrication of membranes of good quality [43]. On the other hand, electrospinning of the 12% solution yielded beaded fibers for the low- and high-voltage settings, while in the middle-voltage setting the resulting membrane had large polymer aggregations connected with each other by fibers. This behavior confirms that a 12% *w*/*v* polymer concentration is lower than optimal, regarding cellulose acetate.

For the 14% *w*/*v* solution, different flow rates were also tried, given that it was the composition that gave the overall optimal results, compared to the 12 and 16% *w*/*v* solutions. A deviation regarding the fiber sizes was observed, namely that the increase in the flow rate brought a decrease in fiber diameter. Theoretically, the opposite should happen, as an increased flow rate leads to the accumulation of a larger quantity of the solution as well as a reduction in the flight time of the jet—this is something that has been reported before [56]. This may happen because the lower flow rate used could be outside the optimal window for this solution. The flow rate has been shown to affect the geometry of the Taylor cone and low flow rates create a thinner, more unstable cone, which can lead to this behavior [57]. At the same time, an inadequate supply of polymer solution to the jet may lead to gaps in the jet which can affect the fiber diameter. At the same time, the Taylor cone may retreat into the nozzle, leading to high variations in fiber sizes [58]. This could also explain the behavior of the 12% *w*/*v* solution, regarding the high inhomogeneity that the three electrospun membranes exhibit.

Although structural stability was a concern, a membrane comprised of misaligned fibers was opted for, as high alignment has been shown to reduce porosity and permeability [59], which could in turn limit the absorbency of the membranes. This was done in an effort to create an interconnected network of pores that would act synergistically to transfer the liquid within the core of the structure, which is something that has been shown to increase flux rate within the structure [60], while also enhancing the capillary forces that promote absorption [61]. Instead, multiple layers were utilized in order to provide sufficient structural stability, but also still be highly absorbent. Moreover, the absorbency presented at this work is generally higher than similar reported works, where CA electrospun membranes present an absorbency of 80–2500% [30,46,62]. At the same time, the reduction in the porosity brought about by the addition of multiple layers, led to a more wettable surface. The weight loss that the fourth and thirteenth electrospun membranes exhibited could be attributed to partial degradation overnight due to hydrolysis of CA, attributed to prolonged immersion. As the structure of the membranes begin to collapse, the release of DI water could occur instead of further absorption [63]. Lastly, for the single-layered membranes, the two samples (1 and 3) with the lowest porosity also showed the lowest absorbency. Porosity has been tied to permeation flux within the work of Wang et al. [64] as well, which can further explain this behavior.

Achieving the desired wettability proved to be challenging. An effort was made to improve the initial wettability of the samples, without compromising their overall functionality, by having them reach their capacity before their intended time of usage. A range of various degrees of hydrophilicity was observed for the electrospun membranes (membranes 2 and 7), with some membranes being slightly hydrophobic, possibly due to the high porosity of the membranes, as previously mentioned, while others exhibited hydrophilicity and quick absorption. However, quick absorption led to a significant decrease in overall absorption capacity—something that could be attributed to air being trapped within the porous system of the membrane. Therefore, a slower absorption mechanism was opted for, in order to provide better longevity to the membrane.

Lastly, the porosity of the membranes was calculated, in an effort to create a multi-layered membrane with a gradient absorption capacity and functionality, depending on the placement of each layer, with a lower-to-higher absorbency planning. The porosity of the electrospun membranes produced within this work was shown to be within reported values for similar works, without being too high, something that could compromise the structural integrity of the membranes. For example, the work of Chumpol et al. [46] suggested a porosity of about 77% for the electrospinning of 7 mL of solution, while Zhou et al. reports a porosity of 87%, but for a solution with a polymer concentration of 8% wt [65]. At the same time, their porosity was significant, ensuring their high absorbency. Another mechanism that affects the porosity appears to be the average fiber size of the electrospun membranes, as it is apparent that when the fiber sizes increase, the porosity decreases—something that has been reported by Capuana et al. as well [66]. The porosity values were also comparable to similar works.

The in vitro degradation showcased the chemical and structural stability of CA, regarding applications that are used for a low amount of time. Moreover, SEM revealed that while the fibers of single-layered membranes return to their initial size after drying, the fibers of the multi-layered membranes are able to retain more liquid for a longer period, thus making this structure superior for applications where liquid absorption and management are paramount.

According to the biological assessments, the membranes fabricated with the different process parameters showed comparable cellular responses. This indicates that differentiation in the fabrication parameters, mainly the variation in voltage and polymer concentration, including mono-layer and multi-layer formats, did not significantly affect (*p* > 0.05) metabolic activity, oxidative stress, or inflammatory signaling. This effect may be attributed to the inherently biocompatible nature of the polymer used and to limited cell–material interactions during the examined exposure period—a behavior that has also been reported for other similarly nanofibrous scaffolds used in biomedical applications [67,68]. Several studies have examined reactive oxygen species (ROS)-related responses in nanofibrous materials using in vitro models [69]. In particular, electrospun polymer nanofiber membranes exhibit ROS-scavenging properties and remain biocompatible in cell-based assays, especially for wound healing applications [70].

Future works should be directed to the implementation of diagnostic devices within the absorbent cores of sanitary pads, in order to lead to accurate and timely diagnosis of diseases that are currently hard to identify. Moreover, mechanical characterization at the next stage of development will be necessary to ensure the membrane’s structural stability. Lastly, the modelling of the electrospinning process based on experimental data could lead to more targeted experiments in the future, limiting the time and effort required for the optimization of each fabrication and providing early estimations for final membrane properties, thus leading to the quicker utilization of specific solution and process parameters according to the target application’s needs.

## 5. Conclusions

The electrospinning of cellulose acetate was investigated in this study. An emphasis was placed on the high voltage that was used, placing the optimal voltage range at 14–18 kV for a 14% *w*/*v* cellulose acetate solution, based on the observed morphological characteristics. At the same, time a middling flow rate appeared to provide the best results regarding fiber size homogeneity.

The absorbency of the electrospun membranes was also evaluated, showing a high absorbency for all samples, which ranged from 5 to 70 times their initial weight for the single-layered membranes, and 15 to 30 times for the multi-layered membranes. A potential mechanism that affects this could be the closing of previously open pores by the addition of layers on top of each other. Adding layers can result in the increase in the membrane’s thickness, which has been shown to reduce the membrane’s porosity.

Based on the measurements conducted on the multi-layered membranes, a three-layered structure was observed to be optimal for the target application, considering absorbency, structural stability and fabrication time, as the fourth layer did not add a significant amount of additional liquid during the absorbency testing. At the same time, ML-2, which showed the fastest absorption rate, only reached about 15 times its initial weight, which was half when compared to the other two multi-layered membranes fabricated. As discussed previously, this may be attributed to air entrapment within the structure that does not allow the liquid to occupy as much space as possible, therefore limiting its volume.

Lastly, the safety assessment of the produced electrospun membranes indicated satisfactory cytocompatibility under the tested in vitro conditions using Hs27 fibroblasts. Further investigations, including long-term and in vivo studies, are needed to confirm broader applicability. Taken together with the favorable absorbency and structural characteristics, these results support the potential of the developed membranes for further biomedical investigation.

## Figures and Tables

**Table 1:** Electrospinning trials.

Polymer Concentration (*w*/*v*)	High Voltage (kV)	Flow Rate (mL/h)	N-C Distance (cm)	Collector RPM	Spinning Time
12%	14	1.2	10	400	5.5 h
	18	1.2	10	400	5.5 h
	22	1.2	10	400	5.5 h
14%	14	0.6	10	400	5.5 h
	14	1.8	10	400	5.5 h
	14	1.2	10	400	5.5 h
	14	1.2	10	400	5.5 h
	18	1.2	10	400	5.5 h
	18	0.6	10	400	5.5 h
	22	1.2	10	400	5.5 h
16%	14	1.2	10	400	5.5 h
	18	1.2	10	400	5.5 h
	22	1.2	h10	400	5.5 h

**Table 2:** Electrospun membrane designation.

Membrane No.	Polymer Concentration (*w*/*v*)	High Voltage (kV)	Flow Rate (mL/h)	Working Distance (cm)	Collector RPM
1	14%	14	1.2	10	400
2	16%	22	1.2	10	400
3	16%	14	1.2	10	400
4	14%	18	1.2	10	400
5	12%	18	1.2	10	400
6	12%	14	1.2	10	400
7	14%	22	1.2	10	400
8	12%	22	1.2	10	400
9	16%	18	1.2	10	400
10	14%	14	0.6	10	400

**Table 3:** Multi-layered membranes as produced from substrate to top layer.

Multi-Layered Membrane No.	Solvent	Concentration (*w*/*v*)	High Voltage (kV)	N-C Distance (cm)	Flow Rate (mL/h)	RPM	Total Spinning Time
1	DMSO/Ac 25/75	16%	14	10	1,2	400	5.5 h
		14%	18	10	1,2	400	5.5 h
		14%	18	10	1,2	400	5.5 h
		12%	14	10	1,2	400	5.5 h
2	DMSO/Ac 25/75	16%	18	10	1,2	400	5.5 h
		14%	22	10	1,2	400	5.5 h
		14%	22	10	1,2	400	5.5 h
		14%	18	10	1,2	400	5.5 h
3	DMSO/Ac 25/75	16%	18	10	1,2	400	5.5 h
		14%	18	10	1,2	400	5.5 h
		14%	18	10	1,2	400	5.5 h

**Table 4:** Standard deviation associated with the high voltage applied and polymer concentration, when all other process parameters remain constant, for a cellulose acetate solution in a solvent system of DMSO/acetone 25/75 *v*/*v*.

High Voltage (kV)	Polymer Concentration *w*/*v* (%)	Average Fiber Diameter (nm)	Standard Deviation
14	12	343.35	35.85%
	14	580.75	20.67%
	16	408.55	25.54%
18	12	319.6	32.74%
	14	348.55	25.90%
	16	543.95	37.14%
22	12	380	30.01%
	14	418.37	33.41%
	16	493.32	31.78%

**Table 5:** Electrospun membranes as they appear in Figure 8.

Electrospun Membrane	Polymer Concentration (*w*/*v*)	High Voltage (kV)	Flow Rate (mL/h)	Working Distance (cm)	Collector RPM
1	14%	14	1.2	10	400
2	16%	22	1.2	10	400
3	16%	14	1.2	10	400
4	14%	18	1.2	10	400
5	12%	18	1.2	10	400
6	12%	14	1.2	10	400
7	14%	22	1.2	10	400
8	12%	22	1.2	10	400
9	16%	18	1.2	10	400
10	14%	14	0.6	10	400
11	14%	14	1.8	10	400
12	14%	14	1.2	15	400
13	14%	18	0.6	10	400

**Table 6:** Electrospun membrane absorption capacity for 3 h and overnight testing.

Electrospun Membrane	Absorption Capacity (3 h, %)	SD (%)	Absorption Capacity (Overnight, %)	SD (%)
1	1951.368	3.23	2157.65	7.56
2	3797.3	20.02	4057.6	19.50
3	440.28	2.19	527.37	15.52
4	7388.21	5.67	7018.2	4.60
5	2088.7	20.70	2358.84	21.48
6	2375.19	3.65	3010.2	13.05
7	2803.77	7.09	3323.83	10.50
8	3885.77	4.59	4254.19	1.39
9	5063.31	3.89	5364.82	4.52
10	4225.97	2.75	4822.8	4.35
11	1834.36	11.98	1933.28	8.18
12	4186.07	8.99	5506.86	4.50
13	3331.92	0.70	3374.87	4.81

**Table 7:** Average thickness and apparent porosity values for each membrane.

Trial	Membrane Thickness (mm)	Porosity (%)
1	0.076 ± 0.0017	67.20 ± 0.29
2	0.1 ± 0.0125	89.38 ± 1.78
3	0.089 ± 0.0021	62.35 ± 0.96
4	0.083 ± 0.0125	91.63 ± 0.93
5	0.076 ± 0.0029	87.81 ± 3.00
6	0.068 ± 0.0019	85.61 ± 0.86
7	0.078 ± 0.0024	82.35 ± 0.76
8	0.064 ± 0.011	85.85 ± 0.94
9	0.107 ± 0.0025	85.32 ± 0.38
10	0.072 ± 0.0016	86.72 ± 1.03
11	0.058 ± 0.0062	86.07 ± 0.28
12	0.056 ± 0.0022	81.56 ± 1.21
13	0.082 ± 0.0033	86.79 ± 0.24

**Table 8:** Average thickness and porosity of the multi-layered membranes.

Multi-Layered Membrane	Thickness (mm)	Porosity (%)
1	0.218 ± 0.023	77.09 ± 0.65
2	0.267 ± 0.005	81.40 ± 0.58
3	0.130 ± 0.01	82.70 ± 0.58

**Table 9:** Contact angle measurements for multi-layered membranes.

Membrane	Initial Contact Angles (°)
ML1	73.07 ± 0.49
ML2	79.56 ± 1.06
ML3	66.20 ± 1.05

## Data Availability

The original contributions presented in this study are included in the article. Further inquiries can be directed to the corresponding author.

## Abbreviations

The following abbreviations are used in this manuscript:

CA	Cellulose acetate
DMSO	Dimethyl sulfoxide
Ac	Acetone
ML	Multi-layered (membrane)
DMEM	Dulbecco’s modified Eagle medium
PBS	Phosphate-buffered saline
FBS	Fetal bovine serum
TNF-a	Tumor necrosis factor-alpha
IL-18	Interleukin 18
